# Peroral choledochoscope in the diagnosis of double common bile duct

**DOI:** 10.1055/a-2830-4046

**Published:** 2026-03-25

**Authors:** Xinghua Peng, Hongfei He, Shaoqiong Cheng, Hua Zhong, Tongxin Liu, Jiaxin Lu, Fang Wang

**Affiliations:** 1 609058 Hepatobiliary Surgery Department 1, Handan First Hospital, Handan, China; 2 609058 Department of Anesthesiology, Handan First Hospital, Handan, China

Double common bile duct is an extremely rare congenital anomaly of the biliary system 1 . We report a case of double common bile duct accompanied by bile duct stones, in which we innovatively applied a peroral choledochoscope (eyeMax Choledochoscope System Digital Controller) to diagnose this biliary malformation.

**Fig. 1:**
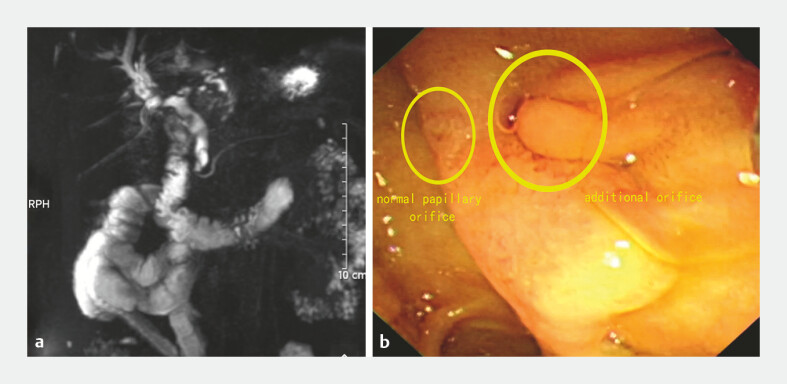
**a** MRCP revealed stones in the right intrahepatic duct and common bile duct, and a duct seemed to be visible near the common bile duct. **b** Duodenoscopy identified an additional orifice to the right of the major duodenal papilla. MRCP, magnetic resonance cholangiopancreatography.

A 60-year-old woman was admitted to our hospital with upper abdominal pain and fever for half a month. The patient underwent left hemihepatectomy for intrahepatic bile duct stones 30 years ago. Magnetic resonance cholangiopancreatography revealed stones in the right intrahepatic duct and common bile duct, and a duct seemed to be visible near the common bile duct ( Fig. 1 **a** ).

**Fig. 2:**
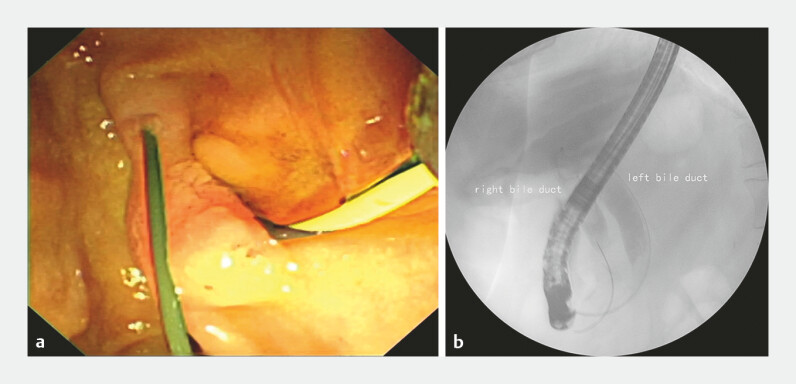
**a** The guide wire was inserted into the common bile duct through the additional orifice and then returns from the duodenal papilla. **b** Cholangiography showed two common bile ducts and the returning guide wire.

**Fig. 3:**
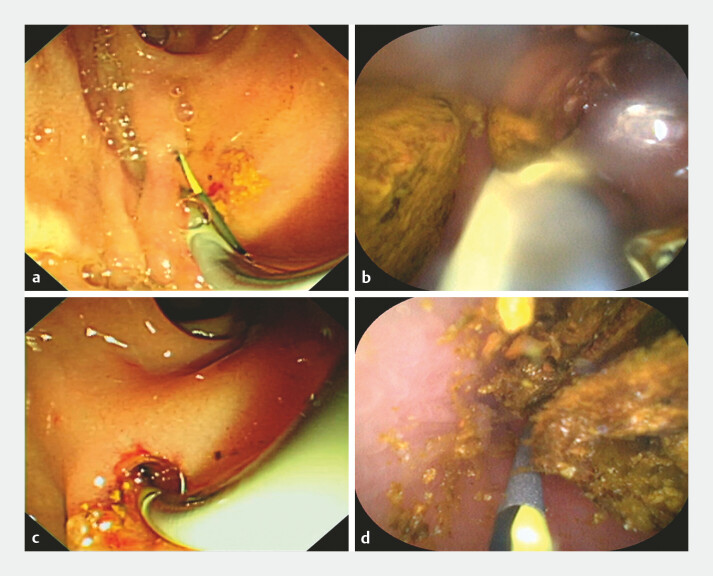
**a, b** Peroral choledochoscopy was performed through the additional orifice, revealing multiple stones in the left common bile duct and intrahepatic bile duct. **c, d** Examination of the right common bile duct via the duodenal papilla also showed normal bile duct mucosa and stones, with the choledochoscope reaching the intrahepatic bile duct.

The patient had cholangitis, and endoscopic retrograde cholangiopancreatography was performed. Duodenoscopy identified an additional orifice to the right of the major duodenal papilla ( Fig. 1 **b** ). The guide wire was inserted into the common bile duct through the additional orifice and then returns from the duodenal papilla ( Fig. 2 **a** ). Cholangiography showed two common bile ducts and the returning guide wire ( Fig. 2 **b** ), and both the bile ducts were dilated with multiple filling defects. When the guide wire was inserted into the duodenal papilla, it can also returns from the the additional orifice. Peroral choledochoscopy was performed through the additional orifice, revealing multiple stones in the left common bile duct and intrahepatic bile duct ( Fig. 3 **a, b** ; Video 1 ). Examination of the right common bile duct via the duodenal papilla also showed normal bile duct mucosa and stones, with the choledochoscope reaching the intrahepatic bile duct ( Fig. 3 **c, d** ). We performed sphincterotomy on the duodenal papilla and water sacexpansion additional orifice; after removing bilateral bile duct stones with basket and balloon, peroral choledochoscopy showed no residual stones in both bile ducts, but the right intrahepatic duct stones cannot be removed completely. The patientʼs postoperative recovery was smooth, and there were no symptoms during the 3-month follow-up. She was advised to undergo follow-up examinations every 6 months.

Peroral choledochoscopy in the diagnosis of double common bile duct.Video 1

Double common bile duct malformation has a high risk of misdiagnosis and may develop biliary cancer 2 3 4 . This is the first attempt to use a peroral choledochoscope for the direct visualization of the bile duct interior and mucosa in this type of disease. The peroral choledochoscope offers a superior level of intraluminal assessment that surpasses conventional imaging and fluoroscopy, improving outcomes for patients with challenging anatomical conditions.

Endoscopy_UCTN_Code_TTT_1AR_2AL Endoscopy_UCTN_Code_CCL_1AZ_2AK
